# An analysis of the vulnerability of informal and formal households to disaster risks in the Rand West City region

**DOI:** 10.4102/jamba.v16i1.1589

**Published:** 2024-05-23

**Authors:** Nomonde Madubula, Elize van Eeden

**Affiliations:** 1 Financial and Fiscal Commission Midrand, Gauteng, South Africa; 2 School of Social Science, Faculty of Humanities, North-West University, Vanderbijlpark, South Africa

**Keywords:** disaster risk, vulnerability, household vulnerability, informal and formal households, mining activities, Bekkersdal

## Abstract

This study aimed to compare how vulnerable informal and formal households are to disaster risks in Bekkersdal mining area in the Rand West City municipality, using a mixed method. A qualitative approach was used to construct a situational analysis of the community, while a questionnaire was used to collect descriptive data. Findings showed both household types (formal and informal) experienced disaster-related risks as their coping resources are limited. Disaster risks such as mining-related incidents (dust, earth tremors and windstorms) are more significant in the informal areas than in formal areas because of their geographical locations. The latter are located in high density areas, with limited access to basic services. Resulting in among others, construction of illegal informal areas and use of illegal electricity connections. These disaster incidents occur against the backdrop of an already vulnerable dolomitic environment that tends to form sinkholes. Thus, the negative impact of mining is the highest disaster risk factor in the area, yet households seem tolerant and distant as they perceive mining as a job opportunity. The study recommended the municipality to include disaster risks in their integrated development plans to ensure that sustainable mining practices are in place to minimise the negative effects in the area. The rehabilitation of mines, measures to prevent informal construction or illegal occupation, and educational awareness on mitigation and adaptation measures are necessary.

**Contribution:**

The study adds to the body of knowledge by revising some old techniques of addressing disaster risk measures, especially in surrounding mining communities.

## Introduction

The article is an attempted response to the main research aim of the study: an in-depth comparative analysis of households’ vulnerabilities to risk-related disasters in formal and informal settings within the Bekkersdal mining community. A disaster risk threatens all those exposed and vulnerable (Inter-American Development Bank 2005; Raheem et al. 2013) and is caused by potential hazardous occurrences and the susceptibility of people to injury or loss. Scholarly debate informs that disaster-related risks should also be understood in terms of people’s level of vulnerability and not merely rely on physical attributes. This is because households who reside in, for example, an informal settlement do so because they are poor, or they do not have the political or economic power to make choices while they are faced with day-to-day urban challenges of unemployment, poverty and the lack of basic services (Abunyewah et al. 2018; McDermotti et al. 2021; Williams et al. 2019). Thus, disaster risks add to the already distressed, poor and vulnerable conditions that households face, which is, among others, a lack of access to basic and financial services (Madhuri et al. 2014).

As in many townships of South Africa, Bekkersdal is in a vulnerable position because of high levels of poverty, poor living standards, unemployment, unequal distribution of assets and ownership, a lack of access to resources, slow growth of the local economy and widespread environmental degradation (COGTA 2005; Rand West City [RWC] 2016–2021). According to Statistics South Africa (STATS SA 2016), formal households are characterised by formal dwellings/houses or brick/concrete block structures on separate stands, yards or on a farm, formal dwellings/houses/rooms/flats in a backyard. On the other hand, informal households are characterised by informal dwellings/shacks in backyards or in informal squatter camps or settlements or on farms. Thus informal households become more vulnerable (the Bekkersdal community being a case in point) because of informal households next to the Donaldson Dam. The dam is known for its mining effluents (acid mine drainage) dating back to over 120 years of mining activities and mine waste, inappropriate land use planning as informal households occupy illegal and unsuitable structures, frequent flooding because of inadequate stormwater management systems and a high frequency of uncontrolled veld fires in the Bekekersdal high density area. They are, therefore, vulnerable to disaster risks because of their disadvantaged position. A study conducted by Sakijege et al. (2012) supports the above arguments. The findings indicated that the lack of stormwater drainage in settlements is the cause of flooding because of densification and poor solid waste management. Furthermore (Coetzee et al. 2016; Failing 2008), note that vulnerability is the extent to which a community or geographical area is likely to be damaged or disrupted by a natural or man-made occurrence because of socioeconomic and political conditions. These conditions either increase the suffering caused by the risk or affect the ability of the community to respond and recover from risks.

The study sought to fill this gap by comparing the vulnerability of informal and formal households to disaster risks in the Bekkersdal area surrounded by mining activities as one size does not fit all. Bekkersdal provides a compelling case study of a localised community’s vulnerability for several reasons. Firstly, the Bekkersdal community is unique in that it is largely located in a dolomite area with many sinkholes and acid mine drainage decanting and spillages. Secondly, the community faces many other challenges, such as high levels of water pollution because of the environmentally damaging mining activities in the area, inappropriate land use planning, frequent flooding because of inadequate stormwater management systems, frequent droughts, acute erosion as a result of unstable soil structures and underlying geological factors, rapid urbanisation and a high frequency of uncontrolled veld fires (Ackerman et al. 2018; Koen et al. 2017 Liefferink 2019; Marais et al. 2018; Van Eeden 2014; West Rand District Municipality [WRDM] 2019). Thus,in understanding the community and household vulnerabilities, the comparison between formal and informal settlements plays a critical role in addressing what the determinants are and what kind of ameliorating measures should be put in place. The introduction encompasses the problem statement and description of the study area and research design. This is followed by the findings, discussion, conclusion and recommendations.

### Problem statement

Global hazardous risks such as climate change impacts, human vulnerability and lack of capacity and intense frequency attributable to rapid urbanisation, settlement in hazardous prone areas (Joshua et al. 2023) are estimated to increase with 70 million people being exposed and R800 million people residing on susceptible areas. South Africa is no exception, as it has been affected by various anthropogenic and natural hazards and disasters of different types and proportions. According to Mulugeta et al. (2007), South Africa is prone to droughts, urban and rural fires, floods and dam failures, earthquakes and sinkholes, epidemics, storms and hazardous waste and materials spillages. Over the years, the South African government has initiated and implemented various programmes and strategies to ameliorate the negative impacts of disaster risks on communities and households. However, the data on which these interventions were based, lacks local information (COGTA 2005; RWC 2016–2021).

The April 2022 floods in KwaZulu-Natal and the Eastern Cape, causing a trail of destruction, attest to rising temperatures. The two provinces reported 435 deaths, displacement of 40 000 people and damage to and destruction of 13 500 houses. The costs in terms of property damage were estimated at over R17 billion (Pinto et al. 2022; South Africa 2022). The World Bank Report (2021) supports the arguments that disasters (such as droughts, floods and storms) in South Africa have, to a great extent, resulted in socioeconomic and environmental losses – in the period between 1900 and 2017, over 100 disastrous events were reported with an estimated death rate of 2200 and 21 million people being displaced and affected.

According to Winde and Stoch (2010), Nealer (2020) and Van Eeden and Khaba (2016), sinkholes are rife in the West Rand area, and they date back to more than 100 years when gold was discovered in the area and water was pumped underground. Infrastructure was poorly managed, creating sinkholes and endangering the delivery of basic services. A study conducted by Winde et al. (2019) on human exposure to uranium during gold mining activities in the Witwatersrand basin, where Westonaria and the town of Bekkersdal are located, found that the levels of concentrated uranium were much higher. Exposure to uranium is a health hazard as it can cause cancer. Liefferink et al. (2017) supported this and pointed to water-related challenges among the informal residents of Bekkersdal. The study found that the water supply in the area was often inadequate or not purified, giving rise to waterborne diseases. In addition, it was found that the Donaldson Dam, which borders the informal households of Bekkersdal, is highly polluted because of mining-related discharges from surrounding areas. Riukulehto (2021) supports the above arguments as households living in the area have become used to mining effluents, tailings dams, sinkholes and dolomite in the West Rand as being an ‘inherent part of their home’ despite it being an alarming and dangerous threat to their livelihood. Dolomites are defined as rock formations that overlie many gold deposits sometimes collapse, causing sinkholes (Ngcobo 2008). Yet, the Donaldson Dam is used by the Bekkersdal community for baptisms and fishing; there are stalls close by where food is sold, and livestock also drink from the dam (Van Eeden 2011).

**FIGURE 1:**
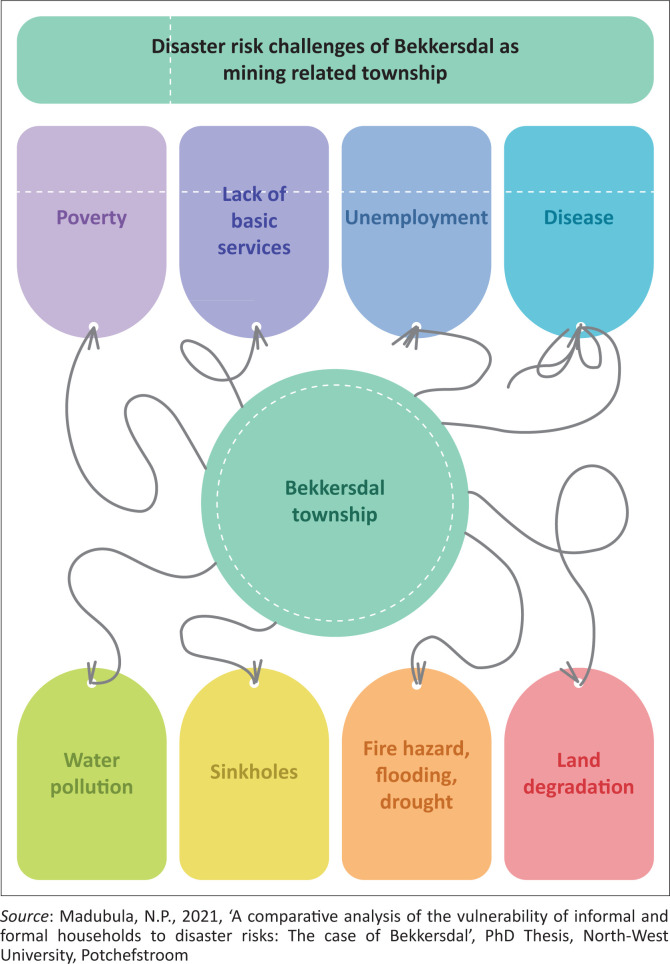
Disaster risk challenges in Bekkersdal.

The 2020–2021 Rand West City (RWC) integrated development plan (IDP) highlighted challenges and threats that municipalities face in its *status quo* analysis. It included poverty and unemployment, an increasing demand for basic services, inadequate infrastructure and maintenance, illegal connection of electricity, pollution, dolomite and sink holes. Figure 1 summarises the main challenges facing the Bekkersdal community, all of which are closely linked to mining activities in the area. Collectively, the foregoing profiles highlight several variables that account for the disaster risks and vulnerability of the Bekkersdal community. All these factors potentially amplify the risks and compromise their vulnerability.

It is critical that interventions should be supported by a robust comparative analysis of community and household vulnerabilities to ensure effective targeting (Ncube et al. 2016; Van Huyssteen et al. 2012). Comparative research has several other advantages, such as offering policymakers a chance to avoid repeating past mistakes and enabling them to save time by targeting resources effectively to deserving groups. Comparative research enables better identification of differences and similarities between different groups and a clearer understanding of the various consequences of interventions, which enables more effective allocation of resources (Fernandez et al. 2006; Gallegos 2011; Jahangiri et al. 2011; Smart 2012). Considering the importance of assessing and comparing the vulnerability of different communities and the drivers of vulnerability, this study undertook a systematic and comprehensive comparative analysis of the vulnerability of informal and formal communities, using Bekkersdal, a unique community surrounded by mining activities, as a case study.

### Study area

**FIGURE 2:**
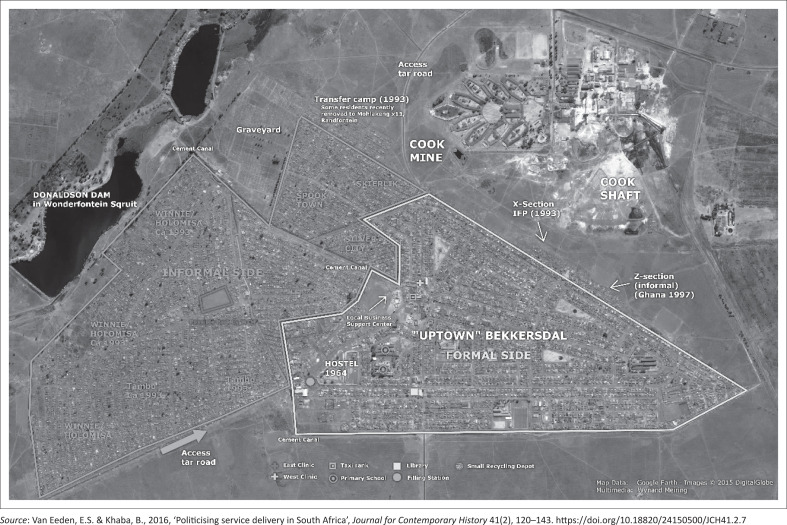
Map of the Bekkersdal Township (formal and informal).

The Bekkersdal township was founded in 1945 as a mining town (De Crom & Nealer 2017), and mining has remained its main economic activity to date. Mining activities created employment opportunities in the area and caused rapid urbanisation (resulting in the establishment of informal areas) and negative environmental impacts (land degradation, pollution and mine effluents). The RWC local municipality, which is the focus of the case study, is in the West Rand District Municipality. The township comprises about 3000 formal and an estimated 13 000 informal households (Liefferink et al. 2017; Municipal IQ 2014). The informal areas include Mandelaville, Winnie/Holomisa, Silver City, Spook Town, the Thambo section and the X section. The formal areas are Proper and Skierlik. The map in Figure 2 provides a pictorial view of informal and formal settlements within Bekkersdal.

## Research methods and design

The study adopted a mixed qualitative and quantitative research approach, using Bekkersdal as a case study to evaluate the vulnerability of formal and informal households to disaster risks in an environmentally and economically vulnerable mining community. Therefore, this study explored quantitative primary survey data and qualitative secondary data from focus group interviews, informal discussions, published literature and policy documents.

The survey in the form of a questionnaire was undertaken to collect data from households’ heads through interviews. Two groups were targeted for the case study. The first respondents were Bekkersdal households from the formal and informal settlements. The second category of respondents were officials from the WRDM, who were responsible for managing disaster risks in the Bekkersdal community of the RWC local municipality. Concerning the first respondents, the questionnaire was used to collect local primary data. Fifteen fieldworkers distributed 16 questionnaires (with subsections) to the households in the formal and informal settlements. Over a period of 3–4 weeks, the field workers interviewed 1000 households (formal and informal), of which, 996 responded. In terms of sample representation, women accounted for 67.3% and 65.7% for formal and informal households, respectively.

The interviews relied mainly on the questionnaire comprising of structured and non-structured questions (representing the quantitative and qualitative approaches, respectively). The questionnaire was administered by a researcher to gain insight on how officials address and deal with disaster risks.

Once the data were collected, the information was ‘cleaned’ and coded. A coding scheme was devised allowing for data to be entered directly into the database. The ‘cleaning’ process entailed identifying and removing outliers, that is, revisiting original questionnaires to address obvious flaws in data entries. Like any survey, there were missing variables, as some respondents did not answer all the questions. As such, questionnaires containing very little data were removed but questionnaires with a small proportion of missing values were retained. The coded information was organised and stored in Excel software. Excel was used for computing descriptive statistics.

### Ethical considerations

An application for full ethical approval was made to Rand West City (acting as gatekeeper) and ethics consent was received 2013 and extended to 2020. The ethics approval number is FH-BE-2013-0014.

This research was undertaken following the strict research guidelines of the North-West University and received ethical clearance and permission to conduct the research as part of the Community Engagement Integrative Multidisciplinary Project related to the Environmental Health and Wellbeing of Mining Communities in Bekkersdal.

## Findings

Key findings of the study below depicted characteristics and variables that relate to households’ vulnerability to disaster risks in the formal and informal areas of Bekkersdal.

### Household characteristics

The distribution of the households sample indicates that 74.1% occupy the informal area, with 25.9% residing in the formal Bekkersdal area, indicating that the majority of the households are susceptible to disaster risks given their geographical location. Further, in terms of sample representation, women accounted for 67.3% and 65.7% in the formal and informal households, respectively. Plausible reason for this could be because of the time the survey was conducted, which was during the day and men were at work.

### Disaster risks in Bekkersdal

A Likert-scale was used to assess disaster risks (droughts, fires, flooding and mining-related incidents) in the formal and informal areas of Bekkersdal. The results in Table 1 indicate that mining-related incidents, social unrest and diseases have been significant in the area. Interestingly, informal sector dwellers cited mining-related incidents (dust and earth tremors) at 60%, followed by social unrest because of service delivery protests at 59%, fires at 51% and sinkholes at 42% as the most significant risks in the area. In the formal areas, however, respondents identified dust and earth tremors, followed by service delivery protests, fires and sinkholes at 31%, 38%, 10% and 5%, respectively. Disease outbreaks (36%), flooding (23%) and windstorms (16%) were recorded as highly significant in the informal areas compared to 41%, 6% and 7% in the formal areas, respectively.

**TABLE 1:** Disaster risks in Bekkersdal.

Disaster risks	Formal (%)			Informal (%)		
	Very significant†	Significant‡	Not significant§	Very significant	Significant	Not significant
Droughts	6.0	24.0	71	12	34	54
Sinkholes	5.0	25.0	70	42	28	31
Windstorms	7.0	36.0	57	16	41	43
Earthquakes	16.3	37.0	46	7	29	64
Mining related incidents (dust, earth tremors)	31.0	28.0	41	60	22	19
Flooding	6.3	26.7	67	23	29	49
Fires	10.0	24.0	66	51	26	23
Social unrest (service delivery protests)	38.0	32.0	30	59	14	28
Health outbreaks (cholera)	41.0	28.0	31	36	19	45
Other, specify	25.0	25.0	50	34	25	42

*Source*: Adapted from Madubula, N.P., 2021, ‘A comparative analysis of the vulnerability of informal and formal households to disaster risks: The case of Bekkersdal’, PhD Thesis, North-West University, Potchefstroom

† Very significant = Impact daily;

‡ Significant = Impact weekly;

§ Not Significant = Impact monthly or seldom.

### Household vulnerability

Households were asked to rate their vulnerability using a Likert scale. As shown in Table 2, children, women, the elderly and people with disabilities and immigrants in the formal and informal sectors, perceived themselves as highly vulnerable. In the informal areas, the scores were the highest; immigrants at 89%, followed by people with disabilities at 87%, children and the elderly at 86% and women at 85%. In the formal areas, children were highly vulnerable at 62%, followed by women 57%, immigrants at 56% and the elderly at 51%, respectively.

**TABLE 2:** Vulnerability levels of household groups.

Household vulnerability groups	Informal (%)			Formal (%)		
	High†	Medium‡	Low§	High	Medium	Low
Children (0–18 years)	86	6	8	62	22	16
Women	85	7	8	57	30	13
Men	6	83	11	17	59	28
Elderly people	86	6	8	51	30	19
People with disabilities	87	6	8	48	26	25
Immigrants	89	3	8	56	20	24

*Source*: Adapted from Madubula, N.P., 2021, ‘A comparative analysis of the vulnerability of informal and formal households to disaster risks: The case of Bekkersdal’, PhD Thesis, North-West University, Potchefstroom

† High vulnerability = households who are in a critical state and would need expert assistance to recover from the impacts of the shocks;

‡ Medium vulnerability = households that require some level of external assistance to overcome the shock;

§ Low vulnerability = households that can adjust to a given shock without needing significant external support.

### Coping measures

Households were asked about the kinds of mechanisms they used to cope with the disaster risks in the area. Findings in Table 3 show that 36% of households in the formal area of Bekkersdal spent cash, only 6% sold assets, 54% received aid, 38% borrowed money and 10% moved elsewhere, compared to the informal area where 35% spent on cash, 15% sold assets, 21% received aid, 30% borrowed money and 32% moved away.

**TABLE 3:** Households’ coping mechanisms.

Household coping mechanisms	Formal (%)			Informal (%)		
	Yes	No	Do not know	Yes	No	Do not know
Spent cash	36	63	0.4	35	52	13
Sold assets	6	93	0.4	15	68	18
Received aid	54	46	1	21	62	17
Borrowed money	38	62	0.4	30	57	12
Move to other places	10	90	0.4	32	55	13

*Source*: Adapted from Madubula, N.P., 2021, ‘A comparative analysis of the vulnerability of informal and formal households to disaster risks: The case of Bekkersdal’, PhD Thesis, North-West University, Potchefstroom

### Waterborne diseases

**FIGURE 3:**
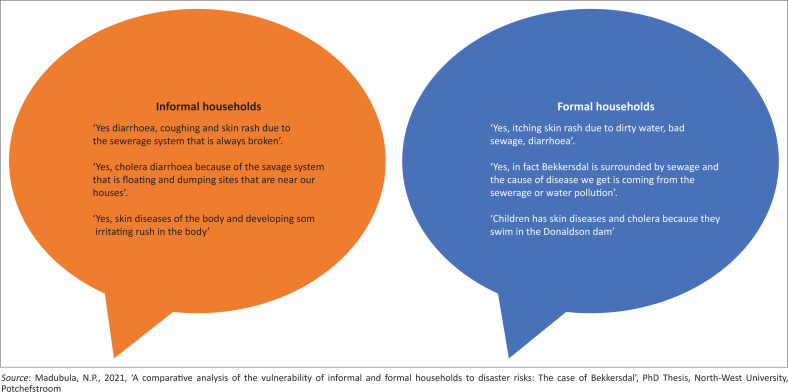
Waterborne diseases.

Figure 3 summarises the study’s findings related to waterborne diseases and direct responses from the households (formal and informal). The findings on waterborne diseases reveal that they affect and infect both households. The symptoms include itchy skin or rash because of broken sewerage and dysfunctional systems and dumping sites near residential areas that cause sickness and pollution.

### Households’ social structures

**FIGURE 4:**
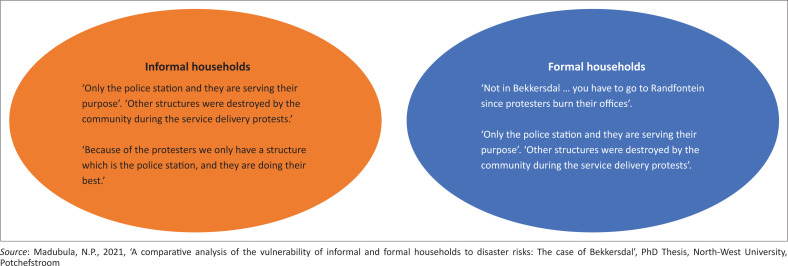
Social structures.

Households in the formal and informal areas were directly probed whether there are any social reporting structures or facilities in place in Bekkersdal that addressed disaster-related risks. The results in Figure 4 show the responses. Both types of households indicated that there are no structures in the Bekkersdal area and facilities were burnt down during social delivery protests. A single police station is used as a reporting facility.

**FIGURE 5:**
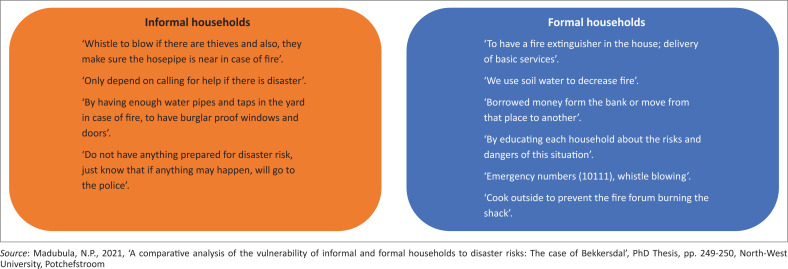
Preparedness and prevention.

### Disaster preparedness and prevention measures

Households were asked to state the type of measures they applied on disaster preparedness and prevention measure to ameliorate the disaster risks. In terms of the findings, 74% of respondents in the formal area indicated that they blow whistles, use hosepipes and report to the police station as preparedness and prevention mechanisms. In the informal areas, 76% mentioned the same measures including using fire extinguishers, moving away from one place to another, using soil water and borrowing money from *mashonisa* [loan sharks].

In corroborating the above results, the disaster practitioner responsible for disaster-related risks in the RWC municipality was probed to indicate the kinds of measures applied by the municipality in mitigating disaster-related impacts and whether these measures are included in the municipality’s IDPs. The disaster practitioner indicated that municipalities use mitigation measures, such as flood management plans, awareness campaigns, community training on basic firefighting, awareness committees, stormwater drainage and flood and fire break plans. These measures are also included in the IDPs of the municipality but only in the formal areas of Bekkersdal. For instance, stormwater drainage exists because proper structures are generally in place. However, the situation is different in the informal areas as there are no structures in place, and drainage systems do not exist. Instead, pit latrines are used.

Further, observations were noted by the field researchers in the study when administering questionnaire to the households. These range from unemployment and poverty in the area, a lack of access to basic services, households’ income solely depending on pension grants, households residing in shacks and the lack of awareness on disaster risks, households living near dumping sites, pollution and households being sickly.

## Discussion

The findings from the descriptive analysis demonstrated the differences in the vulnerability levels of both households in relation to disaster risks. For instance, household distribution by type shows that informal areas are more prone to disaster-related risks than their counterparts in formal areas, mainly because of their geographical location. This includes the structures in the informal area (shacks) compared to the formal areas containing brick houses. There are various reasons for this, such as mining opportunities that influenced the surge in informal houses as a result of the inability of the local government to develop the Bekkersdal area, because environmental risks (dolomite) in the area and the closing of some mining companies that encouraged migrant labour.

Literature also attests to mining activities in Bekkersdal having greatly contributed to the various disaster-related risks (Koen et al. 2017; Liefferink 2019; Mathende et al. 2019). For instance, protests, especially in the informal areas, have been rife because of the lack of service delivery by virtue of the township being in an unplanned and unsafe area (dolomite and sinkholes). Yet, the lack of service delivery should become a secondary risk, with negative mining impacts being a primary risk factor in Bekkersdal (Van Eeden & Riukulehto 2023). From the findings above, both household groups are vulnerable to disaster risks as suggested in the literature (Madubula 2021; Nazari & Keshavar 2023). In fact, immigrants were the most vulnerable groups given that Bekkersdal is a mining area, and people moved to South Africa in search for employment on the mines – an indication that when disaster risks strike, immigrants should be prioritised, followed by people with disabilities, and children and women, respectively.

Informal and formal household coping measures indicate that these households have very little options at their disposal. They spend the bare minimum, have no assets to sell to cushion the effects or even relocate. The impact is greater on the informal households than on the formal structures. Because of Bekkersdal being closer to Donaldson Dam known for its high levels of mine chemicals and radioactive materials (Liefferink et al. 2017), both households were affected and infected by water-borne diseases. The impact on informal households is alarming given that they reside adjacent to the dam. On top of that, because of limited access to water services, especially by informal households, they use Donaldson Dam water as an alternative, mainly for washing clothes, irrigation and recreational purpose; hence they are affected and infected by water-borne diseases.

In terms of social structures being in place, both households agreed that there are no social structures in place for disaster risks or facilities where they could report these incidents. Clinics, schools, churches and police stations in Bekkersdal had to be used. In the absence of any services to assist in reducing the impact of disaster risks, both households are extremely vulnerable. Scholarly debates state that disaster preparedness and preventative measures tend to reduce negative disaster impacts and are key in addressing disaster risks such as awareness, preparedness and early warning systems. However, both households mentioned the use of hosepipes, fire extinguisher and blowing whistles as measures they use to reduce the disaster risks impacts. Additionally, informal households mentioned moving from one place to the other or borrowing money as other preventative measures. According to literature, these are merely response measures. A clear need for the disaster planning, prevention and awareness measures to be in place to reduce and cushion household from the disaster risks impacts.

Probing the disaster risk official responsible for disaster risks in RWC in Bekkersdal about mitigation measures clearly indicated fragmentation between theory and practice. He indicated that mitigation plans exist only ‘in theory’ for the informal households of Bekkersdal. This is because the area is not suitable for residential purposes (inappropriate and unstructured land) where access and availability of basic services are limited and nearly non-existent (because of dolomitic land). Thus, according to Coetzee (2013), the informal area of Bekkersdal has been described as a ‘ticking bomb about to explode to a disaster’. The challenges and opportunities indicated by the disaster risk official on RWC represent and highlight the need for disaster risk mitigation measures to be integrated into IDPs, so that they may be planned, budgeted for and executed as per the plans. Emerging themes from the observations of the study were noted, and can be categorised as being gloomy, the lack of social cohesion and hopelessness. Thus, requiring a need for urgent intervention through a multidisciplinary approach.

## Conclusion

In analysing the vulnerability of both households (formal and informal) to disaster risks in Bekkersdal, the results showed that both households are affected; however, there are some differences that can be attributed to, among others, the different socioeconomic and environmental contexts of households as indicated above. The study’s findings highlighted that the type of housing structure (informal versus formal) has a severe bearing on the vulnerability to disaster risks. In the informal areas, immigrants were the hardest hit by disaster risks, while in the formal areas it was the children. The informal areas in Bekkersdal came about as a result of people moving into the area in search of employment on the mines and subsequently built shacks closer to the mines.

Informal household structures built on dolomite face even more significant disaster risks such as sinkholes, dust, tremors, service delivery protests, fires, disease outbreaks and flooding, than in the formal areas. However, in the case of Bekkersdal’s informal area, it can be argued that the constant service delivery protests are because of poor access and absence of basic services. Mining-related risks, such as the omnipresent dolomite have caused the dire situation, making it impossible to cope effectively and implement mitigating measures and improve infrastructure necessary for a habitable environment. These findings corroborate the reply of the Bekkersdal official when asked if there are any measures in place for disaster risks, for informal households, that is, ‘it exists only in theory’. The lack of social structures on disaster risk in both types of households highlights their vulnerability and the need for the municipality to establish these structures, to lessen the impact on them.

The study recommends that municipalities should stipulate in their IDPs, how they intend to address occupation of households in informal areas that are illegal and unsafe and include awareness campaigns on the dangers of households residing on dolomitic land. Disaster risk officials at RWC should integrate disaster risks (including mining risks) into the IDPs to facilitate budgeting and implementation.

Municipalities should establish permanent social structures to manage mitigation measures as they are one of the coping mechanisms that could be valuable to reduce the vulnerability of households to disaster risks.

Households and communities in Bekkersdal should be made aware of and educated in basic disaster risk management. This should include the real reasons for the limited or non-delivery of basic services in the area, which is largely attributable to the mining risks and making it impossible to develop the area further for basic services’ infrastructure. By-laws on mining disasters should be enforced by municipal disaster officials with penalties imposed for noncompliance, as well as the rehabilitation of mines so as to promote sustainable measures of mining.
